# Epigenetic Regulation in Sepsis, Role in Pathophysiology and Therapeutic Perspective

**DOI:** 10.3389/fmed.2021.685333

**Published:** 2021-07-12

**Authors:** Renata Brito Falcão-Holanda, Milena Karina Colo Brunialti, Miriam Galvonas Jasiulionis, Reinaldo Salomão

**Affiliations:** ^1^Division of Infectious Diseases, Escola Paulista de Medicina, Universidade Federal de São Paulo, São Paulo, Brazil; ^2^Department of Pharmacology, Escola Paulista de Medicina, Universidade Federal de São Paulo, São Paulo, Brazil

**Keywords:** sepsis, epigenetics, chromatin remodeling, DNA methylation, histone modification

## Abstract

Sepsis is characterized by an initial hyperinflammatory response, with intense cell activation and cytokine storm. In parallel, a prolonged compensatory anti-inflammatory response, known as immunological tolerance, can lead to immunosuppression. Clinically, this condition is associated with multiple organ failure, resulting in the patient's death. The mechanisms underlying the pathophysiology of sepsis are not yet fully understood, but evidence is strong showing that epigenetic changes, including DNA methylation and post-translational modifications of histones, modulate the inflammatory response of sepsis. During the onset of infection, host cells undergo epigenetic changes that favor pathogen survival. Besides, epigenetic changes in essential genes also orchestrate the patient's inflammatory response. In this review, we gathered studies on sepsis and epigenetics to show the central role of epigenetic mechanisms in various aspects of the pathogenesis of sepsis and the potential of epigenetic interventions for its treatment.

## Introduction

Sepsis is a syndrome that includes different abnormalities, described in 1992 as systemic inflammatory response syndrome. It was believed that its pathogenesis was mainly due to an unbalanced inflammatory response of the organism triggered by the presence of an infectious agent. This response is much more complex is characterized by the simultaneous exacerbation of inflammatory, metabolic, catabolic, and immunosuppressive pathways, with lingering effects and difficulty in restoring basal homeostasis (1, 2). The concept of sepsis and the understanding of its pathogenesis are continually evolving. Many of the changes considered a dysregulated host response to infection may be, at least in part, an effort to adapt to a hostile environment (3).

Despite all efforts to unravel the mechanisms that orchestrate sepsis, questions remain about its pathophysiology. Epigenetic mechanisms play a prominent role in regulating gene transcription, and gene transcription undergoes significant changes during sepsis. Therefore, epigenetic mechanisms are involved in the acute events of sepsis and in the long-standing post-septic effects on the host response.

## Definition OF Epigenetic Mechanisms

Epigenetic changes are described in literature as chemical changes in chromatin, inherited during cell division, with a role in regulating gene expression and genome stability, without involving changes in the DNA sequence (4). The most studied epigenetic mechanisms are DNA methylation and post-translational modifications (PTMs) of histones but also include changes in chromatin remodeling and regulation by non-coding RNAs (ncRNA) (5). Information on the epigenetic changes plays an important role in regulating DNA processes, such as transcription, repair, and replication. As a result, abnormal expression patterns of gene changes in chromatin regulators may have discrepant results (6).

**Figure 1:**
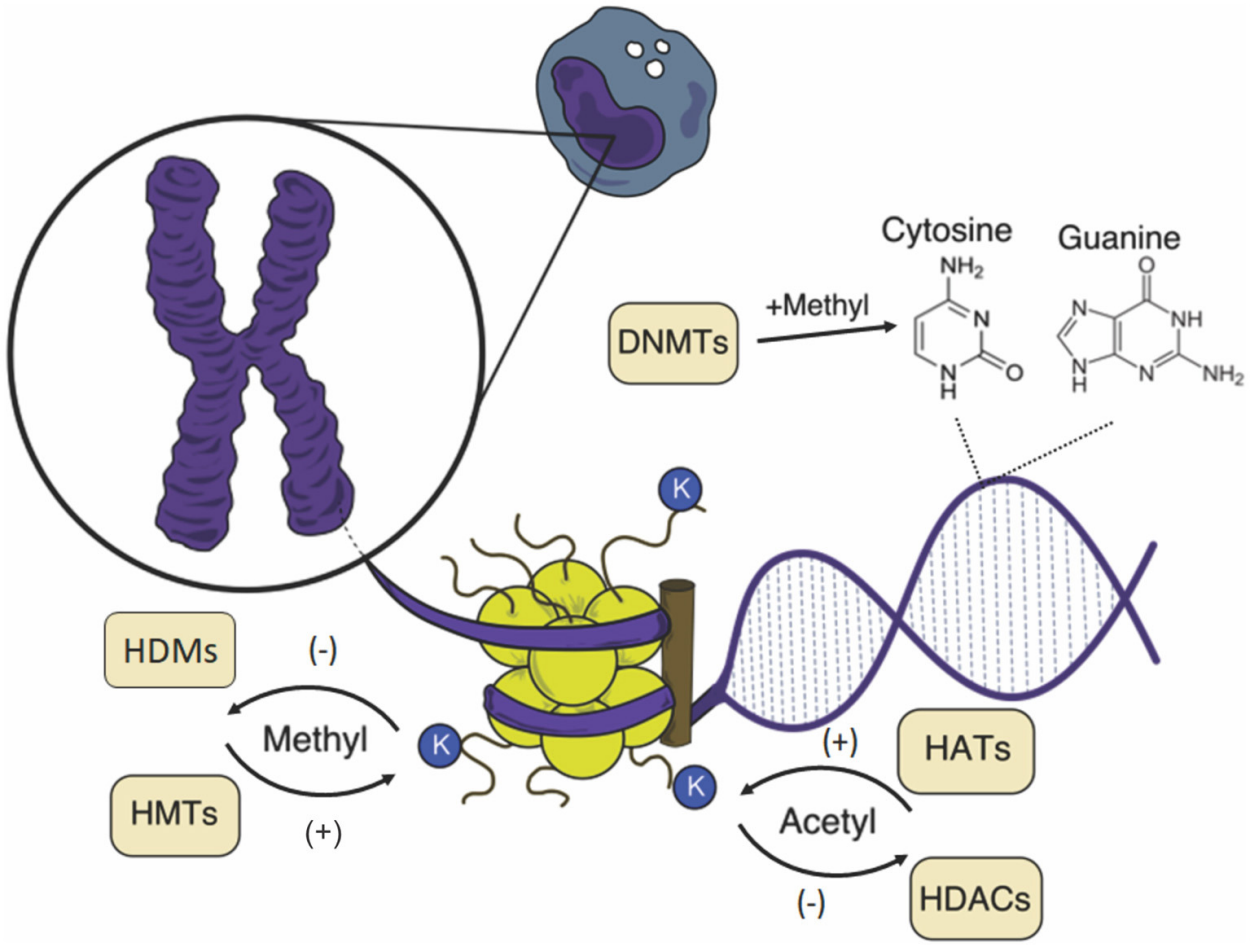
Schematic representation of epigenetic changes in the mononuclear cell. The chromosome is composed of chromatin, a complex formed by DNA and nucleosomes, and the core is formed by an octamer of histones. Both DNA and histones can suffer the action of catalyzing enzymes of chemical groups that influence the chromatin structure, affecting gene expression. K, lysine; HATs, histone acetyltransferases; HDACs, histone deacetylases; HMTs, histone methyltransferases; HDMs, histone demethylases; DNMTs, DNA methyltransferases.

Gene activation or silencing is controlled by enzymes that add or remove chemical groups (acetyl, methyl, among others) in chromatin (Figure 1). These modifications interact with “reader” proteins that have unique structurally conserved domains present in various chromatin regulators and transcription factors, recruiting components of transcriptional machinery and chromatin remodeling complexes (6). These complexes can be subdivided into two main regions: heterochromatin, composed mainly of inactive genes, with late and highly condensed replication; and euchromatin, which contains most of the active genes and has the loosest chromatin (7).

DNA methylation and histone modifications are complementary dynamic processes that together determine the pattern of gene expression, essential in the development, differentiation, and cellular function (8); from the beginning of development and throughout an individual's life, they act regularly and physiologically. Epigenetic marks have plasticity in response to the cellular state and the environment. Epigenetic patterns are influenced by environmental factors during pregnancy, neonatal phase, puberty, and adulthood, and even by exposure to radiation and other chemical and physical agents. In addition, epigenetic errors are associated with the development of chronic diseases in humans (9, 10).

### DNA Methylation

In mammals, DNA methylation occurs predominantly in cytosines that precede guanine, called CpG dinucleotides. DNA methyltransferases (DNMTs) are enzymes that catalyze the transfer of the methyl group (–CH_3_) to carbon 5 of the cytosine, converting it to 5-methylcytosine (5mC) (11). DNMT1 is a maintenance methyltransferase that maintains the mitotic inheritance of the DNA bases through the preferential recognition of hemimethylated DNA during replication, methylating the newly synthesized CpG dinucleotides, generating two new methylated DNA molecules (12). DNMT3a and DNMT3b can recognize any strand of unmethylated DNA and act mainly in establishing new methylation patterns, playing a fundamental role during embryogenesis (13).

More than half of all genes contain high concentrations of CpGs (CpG islands) in their promoters. Gene's promoters containing unmethylated CpGs give the gene a permissive state for transcription. In contrast, hypermethylation of these promoters may prevent binding of transcriptional factors and/or recruiting methyl-binding proteins and repressor complexes, resulting in gene silencing (14).

### Histone Modification

Histones are proteins that compose nucleosomes H1, H2A, H2B, H3, and H4. They have amino acid residues, mainly in their N-terminal portions, subject to covalent modifications, such as acetylation, phosphorylation, methylation, and ubiquitination, which regulate the chromatin structure. Histone modifications can either modify their load or recruit proteins and complexes that affect the transcription of genes present in the region, DNA repair, and replication (6, 15).

Among these modifications, the acetylation of lysines in the N-terminal portions of histones is dynamic and catalyzed by histone acetyltransferase enzymes (HATs). Addition of acetyl groups neutralizes the positive charge of the lysine, weakening the electrostatic interaction between histones and negatively charged DNA, which favors transcriptional activation. Another family of enzymes that is also part of this process is histone deacetylases (HDACs), which have opposite effects to HATs and remove the acetyl group, restoring the positive charge of lysine (15).

Histone methylation occurs mainly in the side chain of lysine and arginine residues through the action of histone methyltransferases (HMTs). Lysines can receive more than one methyl group so that gene transcription can be suppressed or activated, depending on the number of methyl groups and the modified amino acid residue (16). In contrast to acetylation, histone methylation does not alter the general charge of the molecule. This modification was once considered static and stable. However, different families of histone demethylases (HDMs) enzymes act on the lysine residues (15, 17).

Table 1 shows the most frequent changes in histones, their function, the enzymes promoting the changes, and location (18–20).

**Table 1:** Effects of the most frequent histone changes in gene transcription.

**Histone modification**	**Modifying enzymes**	**Function**	**Location**
H3K4me1	SET1, SET7/9, MLL, SMYD2, PRDM9	Activation	Enhancers
H3K4me3	SET1, MLL1, MLL2, SMYD3, PRDM9	Activation	Promoters
H3K9ac	GCN5	Activation	Enhancers, promoters
H3K27ac	GCN5	Activation	Enhancers, promoters
H3K27me3	EZH1, EZH2	Repression	Promoters, gene-rich regions
H3K9me3	SUV39H1, SUV39H2	Repression	Satellite repeats, telomeres, pericentromeres

*From references From references (18–20)*.

### Non-coding RNAs

In addition to the classic epigenetic mechanisms of DNA and histones, a new layer of complexity involving non-coding RNAs has emerged as an important post-transcriptional regulator of gene expression (21). Thus, ncRNAs are a group of RNAs that do not encode functional proteins, being broadly classified as short (<200 nucleotides) or long (more than 200 nucleotides), and these can be grouped by their genomic origin and biogenic processes (22).

MicroRNAs (miRNAs) belong to the most studied and highly conserved class of short ncRNAs, presenting 19–22 nucleotides (nt) in length, which destabilize messenger RNA (mRNA) by binding to 3′ untranslated regions (3′-UTR) or inhibiting protein translation (23, 24). In contrast, long ncRNAs (lncRNAs), generally not much conserved among species, have a multitude of roles, including gene expression regulation at epigenetic, transcriptional, and post-transcriptional levels (23, 25).

## Epigenetic Regulation of the Immune System

**Figure 2:**
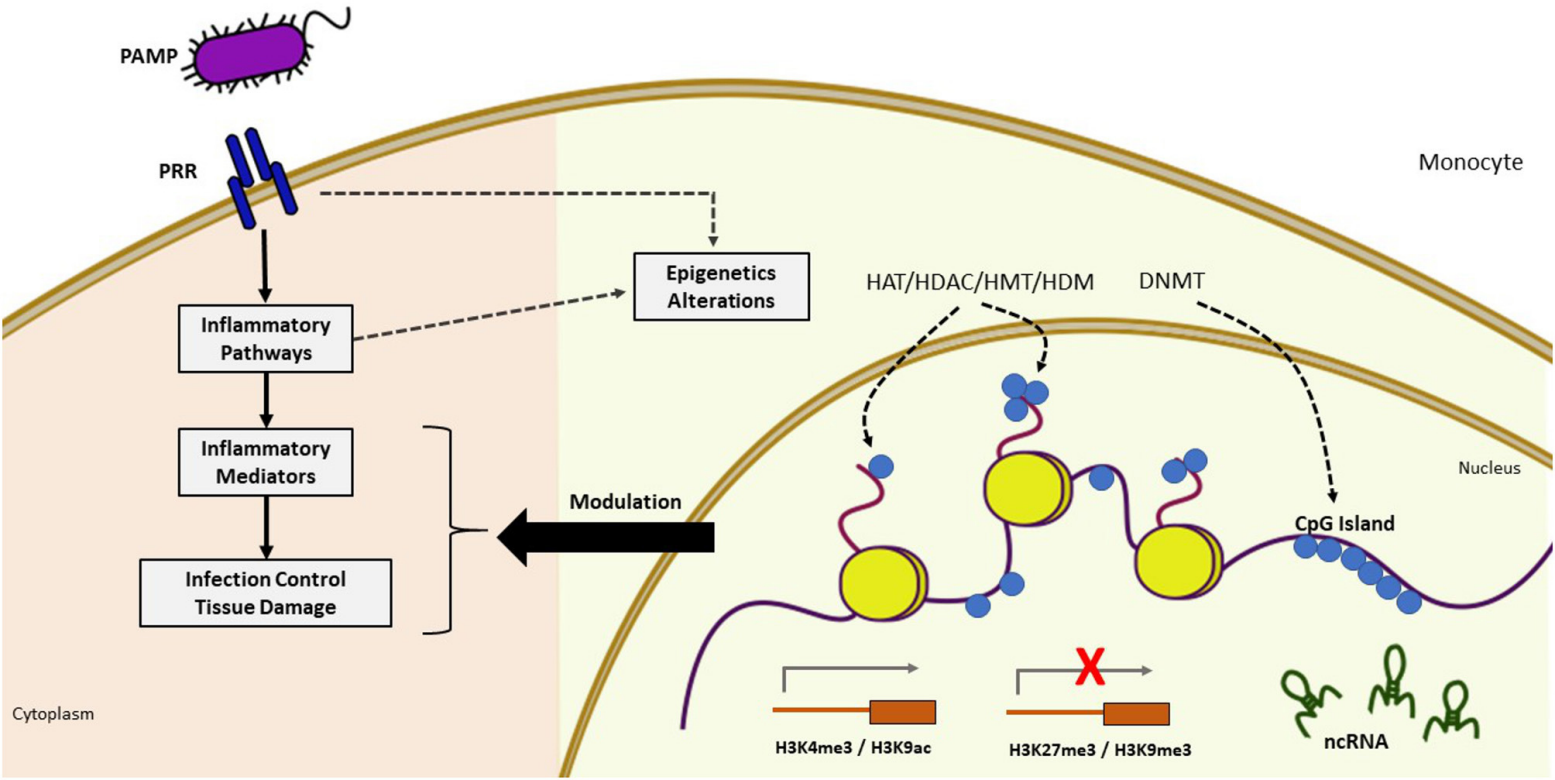
Epigenetic modifications during infection. Pathogen recognition by PRR triggers cascades of intracellular signaling activating inflammatory mediators which induces dynamic changes in chromatin through epigenetic mechanisms, leading to increased or decreased gene activation. The pathogen can also directly alter chromatin through the influence of epigenetic modifiers. These epigenetic alterations (yellow background) may modulate the inflammatory response (pink background). Sepsis induces profound changes in gene expression involved in the inflammatory process and host defense. Epigenetic modifications play a central role in its regulation as evidenced by the presence of differentially methylated CpG islands, several modifications of histones with effects on gene activation (H3K4me, H3K9ac) and repression (H3K27me3, H3K9me3), and the presence of differentially expressed ncRNAs. PAMP, pathogen-associated molecular pattern; PRR, pattern recognition receptor; DNMT, DNA methyltransferase; HAT, histone acetyltransferase; HDAC, histone deacetylase; HDM, histone demethylase; HMT, histone methyltransferases; ncRNA, non-coding RNA.

The immune system can recognize different agents and substances foreign to the body, triggering an immune response mediated by immediate reactions of innate immunity and late responses of adaptive immunity through signaling pathways that are strictly regulated at different levels. Epigenetic changes can also occur during an infectious process, so changes in the epigenome can affect the immune cell phenotype, interfering with the response to infection and contributing to inflammatory disorders (Figure 2) (26–28).

During sepsis, the host innate immune system cells release an excessive number of inflammatory mediators through recognizing the pathogen by pattern recognition receptors (PRRs) that identify the microorganism through pathogen-associated molecular patterns (PAMPs) and damages (DAMPs). These include Toll-like receptors (TLRs), cytosolic RIG-I-like receptors (RLRs), NOD-like receptors (NLRs), and C-type lectin receptors (CLRs), which induce complex intracellular signaling with complementary activities that activate transcriptional factors that regulate inflammatory response genes, generating dynamic changes in chromatin (26, 29–31).

Pathogens are capable of various epigenetic strategies to guarantee their survival and replication, in such a way that they decrease PRR detection and signaling pathways and modulate the expression of immunity-activating and -repressing substances. Thus, a chronic infection can induce epigenetic dysregulation, contributing to the pathogenesis of infectious diseases and even cancer. However, considering that epigenetic changes are potentially reversible, these could be reversed, allowing the host immunity to return to respond efficiently to stimuli (27).

The lipopolysaccharide (LPS) present in the cell wall of gram-negative bacteria binds to TLR-4 receptor, inducing the expression of several genes via the transcription factor NF-κB, such as tumor necrosis factor (TNF), interleukin 1 (IL-1), and IL-8. This activation generates a local and systemic inflammatory process, resulting in coagulation, vasodilation, endothelial escape, scrolling, and leakage of neutrophils and inflammatory mediators to the extravascular space, which can lead to organ dysfunction and hypotension (2, 3, 30–32). LPS stimulation in human monocytes results in the erasure of a repression marker, histone methylation into lysine 9 (H3K9me) in inducible inflammatory gene promoters, regulating these genes (33). In the human endotoxin model, transcriptome analysis revealed that 3,714 genes undergo transcriptional changes after 2 h of exposure, with changes in DNA methylation in several regions of the genome, correlating these results with the tolerance of the immune system and the increase in vulnerability to subsequent infections (34, 35).

Recent evidence has shown that the innate immune system can generate an immune memory mediated by epigenetic reprogramming of transcription pathways, known as trained immunity. This consists of the functional long-lasting reprogramming of innate immune cells in response to exogenous or endogenous stimuli, generating an altered response to a second challenge after returning to baseline (36). For example, individuals vaccinated with BCG (Bacille Calmette-Guérin) have monocyte epigenetic reprogramming throughout the genome, with increased H3K4 trimethylation activation mark (me3), increasing IL-1β production and protection against viral infections in an experimental model of yellow fever. These functional changes indicate trained immunity (37).

During sepsis, a phenomenon known as immunological tolerance occurs. The immune system of patients leaves the state of hyper-inflammation, called a cytokine storm, and goes to a dysfunctional state, where the innate cells do not respond adequately to posterior stimuli (3). In this process, there is a reorganization of the immune functions and metabolic processes of inflammatory cells, with suppression to subsequent challenges as part of this acute cellular reprogramming. Studies show epigenetic modifications are essential for establishing immunosuppression in late sepsis. These modifications include changes in histone marks, loss of activation marks in promoter regions, and gene enhancers that are negatively transcribed into tolerant monocytes (2, 34, 38, 39).

In a model of tolerance induced by LPS, nuclear factor-κB (NF-kB)-activated genes are downregulated. In contrast, genes related to the p38 pathway are preserved, showing a different regulation from the TLR cascades during immunoparalysis (40). Austenaa and colleagues showed that the H3K4me3 epigenetic activation mark participates in regulating the TLR4 signaling pathway and described the profile of this modification in the mouse macrophage genome during the response to LPS (41).

T cells recognize antigens through the human leukocyte antigen (HLA) system [the major histocompatibility complex (MHC) in humans] that are expressed on the surface of host cells; research points to a decrease in the expression of HLA-DR in septic monocytes and DCs (2, 42, 43), combined with transcription reduction of the class II transactivator gene (CIITA), which is modulated by the action of HATs (44, 45).

In this context, the dysregulation of innate and adaptive immunity is associated with harmful consequences, which, together with organ failure, lead to increased short- and long-term mortality in septic patients (46).

Another fundamental mechanism for regulating the inflammatory response is cellular metabolism. During sepsis, innate immunity cells activate a series of intracellular cascades that result in cellular metabolism alterations. The metabolic shift from oxidative phosphorylation to glycolysis during acute inflammation provides the necessary energy for cell function and induces an accumulation of metabolites that function as cofactors of epigenetic enzymes (47).

Thus, a reduction in intracellular levels of acetyl-CoA can decrease histone acetylation. The accumulation of nicotinamide adenine dinucleotide (NAD)+ activates histone deacetylases of the sirtuin class, leading to lower acetylation levels. High concentrations of fumarate inhibit the histone demethylase enzyme KDM5, responsible for removing methyl groups. Therefore, several cellular metabolites can activate or inhibit different enzymes involved in epigenetic programming. They induce changes in chromatin and DNA, modulate gene transcription, and lead to different functional states during sepsis, such as excessive inflammation immunoparalysis (2, 33).

## Epigenetic Regulation in Sepsis

Different epigenetic changes have already been associated with immune activation and tolerance during sepsis, contributing to the process of prolonged inflammation, organ failure, persistent immunosuppression, development of severe secondary infections, and even death (36, 48, 49).

Much of the research that correlates epigenetics and sepsis has been with *in vitro* studies or animal models, with scarce data in septic patients (Tables 2, 3).

**Table 2:** *In vitro* and *in vivo* experimental studies evaluating epigenetic modifications in LPS challenge and infection.

**Study**	**Epigenetic modification**	**Experimental model**	**Results**
***In vitro***			
(41)	Histone methylation	Macrophages of Wbp7 –/– mice exposed to LPS	Macrophages Wbp7 –/– show impaired responses to LPS, with loss of H3K4me3
(50)	Histone methylation histone acetylation	BMM stimulated with LPS	Epigenetic changes are associated with silencing of inflammatory genes and priming of antimicrobial effector
(51)	Histone methylation	Murine RAW264.7 cells and BMDMs upon LPS stimulation	LPS stimulation resulted in enhanced methylation at H3K4 and H3K9 in cells
(52)	Histone methylation	Raw264.7 macrophages LPS-treated	The JmjC-Jmjd3 domain protein is H3K27me macrophage-induced demethylase in the presence of bacterial products and inflammatory cytokines
(53)	Histone methylation	BMM stimulated with LPS	Jmjd3 interferes with the transcription of LPS-activated genes in an independent way to demethylate H3K27
***In vivo***			
(54)	Histone acetylation	ALI sepsis in murine	ALI sepsis reduces the levels of histone H3 lysine acetylation that permits the transcription of angiogenic genes in the lung, kidney, and liver
(55)	DNA methylation	ALI sepsis in rat	1,721 genes had aberrant methylation in the rat's lung tissue with acute LPS-induced injury
(56)	Histone acetylation DNA methylation	ALI sepsis in mice	Combined treatment of DNMTi and HDACi alleviates inflammation-induced pyroptosis and apoptosis during ALI
(57)	Histone acetylation	CLP-induced sepsis in mice	Pretreatment with HDACi 30 min before CLP resulted in decreased lung injury and increased survival
(58)	DNA methylation	CLP-induced sepsis in mice	Treatment with decitabine reduces DNMTs, minimizes NF-kB activation, and attenuates inflammatory cytokine levels, inhibiting sepsis progression
(59)	DNA methylation	Rat model of endotoxemia	Treatment with DMNTi (procainamide) reduced the levels of DMNT1 and 5-methylcytosine, improving inflammatory infiltrate and superoxide production in the lung
(60)	Histone acetylation	Mice injected with LPS	Prophylactic treatment with HDACi (SAHA) reduced levels of TNF-α, IL-1-β, IL-6, and IFN-γ induced by LPS
(61)	Histone acetylation	Mice injected with LPS	SAHA-treated mice had increased survival than untreated mice

*LPS, lipopolysaccharide; BMM, bone marrow macrophage; BMDM, bone marrow–derived macrophage; ALI, acute lung injury–induced sepsis; DMNTi, DNA methyltransferase inhibitor; HDACi, histone deacetylase inhibitor; CLP, cecal ligation and puncture; SAHA, suberoylanilide hydroxamic acid; DMNT1, DNA methyltransferase 1; JMJD3, Jumonji domain-containing protein D3*.

**Table 3:** Epigenetic modifications in human cells *in vitro* and in different clinical settings.

**Study**	**Epigenetic modification**	**Model**	**Results**
(33)	Histone methylation	Human monocytes exposed to LPS	Exposure to LPS changed the methylation pattern of H3K9 in a set of inflammatory gene
(34)	DNA methylation histone methylation	Human monocytes exposed to LPS	Exposure to endotoxin generated changes in DNA methylation, mainly demethylation, and a gain of acetyl in H3K27 and methyl H3K4 in cytokine promoters
(62)	Histone acetylation	Human monocyte cell model of endotoxin tolerance	SIRT1 coordinates the epigenetic and bioenergy shifts
(63)	DNA methylation histone methylation	Human monocyte cell line THP-1 incubated with LPS	In tolerant macrophages, the interaction of DNA methylation with H3K9 methylation silences TNF-α expression
(64)	Histone methylation histone acetylation	Monocytes from septic patients	Sepsis induces changes in chromatin, with selective and precise changes in promoter regions of immunological genes
(65)	DNA methylation	Adults patients withSepsis	The DNA methylation profile showed 668 differentially methylated regions between patients with sepsis and patients with critical non-septic diseases
(66)	DNA methylation histone methylation	Adult patients with community-acquired pneumonia	Chromatin remodeling occurs in community-acquired pneumonia associated with extensive transcriptional deregulation of chromatin-modifying enzymes
(67)	DNA methylation	Neonates with bacterial sepsis	Analysis of the entire epigenome of whole blood samples reveals 81 differently methylated CpG sites in 64 genes, where functional analysis showed an enrichment of protocadherin genes in neonatal sepsis

*LPS, lipopolysaccharide; SIRT1, sirtuin 1*.

### Histone Modification and Sepsis

Foster and collaborators presented the first evidence linking tolerance to LPS with epigenetic mechanisms. They showed that in mouse macrophages, a different response occurs in genes induced by TLR4. These responses were divided into two classes: class T composed of pro-inflammatory genes, which were inhibited in tolerant macrophages; and the NT class genes, composed of antimicrobials that were not inhibited in these macrophages. In the promoters of inflammatory genes, the H3K4me3 activation marks and H4 acetylation were lost during a re-exposition to LPS, and the NT class genes remained with the presence of the activation marks after a second challenge (50). Also, monocytes exposed to LPS do not show active histone marks in the promoter region and in gene enhancers that participate in lipid metabolism and phagocytic pathways, resulting in transcriptional inactivity of these genes through new stimulus (31).

In human sepsis, selective and precise changes in chromatin occur in regulatory regions of genes that participate in the immune process. Chromatin immunoprecipitation combined with high-throughput sequencing showed that in the cells of septic patients, transcriptional activation marks (H3K4me3 and H3K9ac) increased in genes related to immune response; in contrast, genes involved in processing and presenting antigens gained the repression mark (H3K27me3) compared with healthy controls (64). Differences in epigenetic marks can be explained by their plasticity at different times of exposure to the pathogen. Zhao and colleagues found the presence of the activation mark H3K4me2 in bone marrow–derived macrophages (BMDMs) in mice after 30 min of stimulation with LPS, with a return to baseline levels after prolonged exposure to the stimulus (51).

Organ dysfunction occurs during sepsis due to the excessive initial response of cytokines that generate tissue damage. In a model of acute lung injury (ALI) induced by sepsis in mice, loss of histone acetylation was observed in promoters of the main angiogenic genes in the lung and extrapulmonary organs. This systemic response of negative transcription regulation has been included in the pathogenesis of microvascular leakage induced by sepsis and multiple organ dysfunction syndrome (MODS) (54), suggesting early intervention can preserve these epigenetic marks, maintaining endothelial integrity (68). Mice pretreated with HDAC inhibitors attenuated ALI during sepsis (57).

### Epigenetic Regulators and Sepsis

The activity of the enzymes HATs and HDACs can be modulated by LPS, but their contribution to endotoxin tolerance is not yet clear. A study showed that the inhibition of acetyl-lysine binding domain, known as bromodomain and its subfamily bromo- and extra-terminal (BET), induces a negative regulation of inflammatory genes in activated macrophages, reducing inflammation in a model of bacterial sepsis in murine (69).

In the NF-kB activation signaling pathway, the CREB-binding protein (CBP)—a transcriptional coactivator of HAT function—contains bromodomains that bind to acetylated histones H3 and H4 in a way that allows the expression of pro-inflammatory cytokine genes (70). Exposure to LPS increases the stability of CBP by reducing interaction with the FBXL19 subunit of ubiquitin ligase 3 and activating the deubiquitylating enzyme USP14, resulting in chromatin remodeling and cytokine gene expression (71).

The expression of the Jumonji domain-containing protein D3 (Jmjd3) enzyme, a H3K27me histone demethylase class, is induced in macrophages by the transcription factor NF-kB in response to LPS, and binds to genes targeting proteins of the Polycomb group, which belongs to the Chromobox family proteins and mediates gene silencing, regulating the levels of the repressor mark H3K27me3 and transcriptional activity, independent of H3K27 demethylation (52, 53).

Cellular bioenergetic changes during sepsis can also be coordinated by epigenetic mechanisms. During sepsis, sirtuin 1 (SIRT1) rapidly accumulates in the TNF-α and IL-1β gene promoters, deacetylating H4K16 and blocking NF-kB-dependent transcription (62). The presence of SIRT6 can also attenuate NF-kB signaling by deacetylating H3K9 in chromatin (72). Besides, in endotoxin tolerance, the interaction of DNA methylation with histone H3K9 methylation silences the expression of some pro-inflammatory genes (63, 73). LPS activates M1 macrophages, which present a high rate of glycolysis, leading to HDAC degradation, which interferes with the activity of inflammatory cytokines (74, 75). α-Ketoglutarate (αKG), a tricarboxylic acid cycle (TCA) intermediate, favors tolerance to endotoxin in inflammatory genes after M1 macrophages are activated, independently of Jmjd3 (76). Exposure to LPS also increases the metabolism of one-carbon, which produces S-adenosyl methionine, a potent methyl donor (77).

### DNA Methylation and Sepsis

A pilot study involving septic and non-septic patients analyzed methylation throughout the genome in the samples of these individuals and found 668 differentially methylated regions (DMRs) between the septic vs. non-septic groups, among which 56 genes have already been associated with sepsis in literature (65). Blood transcriptome analysis of patients with community-acquired pneumonia identified several chromatin-modifying enzymes are differentially expressed in the initial sepsis, leading to chromatin reorganization and stimulating widespread transcriptional reprogramming (66).

Retrospective research evaluated whether the DNA methylation pattern of CpG sites in the procalcitonin gene [polypeptide related to α calcitonin (CALCA)] could be used as an epigenetic biomarker for bacterial sepsis in premature newborns. These preterm patients showed variation in the DNA methylation status of the CALCA promoter in different types of bacterial sepsis, suggesting different regulation of this gene at the epigenetic level according to the type of infection (78). In a further approach searching for prognostic markers of neonatal sepsis, a small epigenome study analyzed the methylation status of CpGs in blood samples from 3 septic neonates and 3 non-septic and found 81 differently methylated CpG sites in 64 genes, whose functional analysis showed the enrichment of protocadherin genes in neonatal sepsis (67).

An experimental model of LPS-induced ALI found an increase in DNMT1 and 5-methylcytosine, accompanied by neutrophil infiltration and superoxide production in the lung tissue of endotoxemic rats (59). Another epigenomic analysis showed aberrant DNA methylation occurs in promoter regions of 1,721 genes, many of which participate in the hyperinflammatory response (55).

### Non-coding RNAs and Sepsis

ncRNAs are also involved in the pathogenesis of sepsis. A study analyzing the co-expression network of protein-coding and lncRNAs in septic and healthy neutrophils showed that several lncRNAs are linked to genes differentially expressed during sepsis and appear to have a regulatory role in the translation of proteins, and participate in regulatory loops that are altered during sepsis (79). They were detected as sepsis regulators because of the interaction between lncRNAs and sepsis co-expression modules identified by whole blood RNA expression profile of septic patients (80). An analysis of the transcriptome in blood leukocytes of volunteers with sepsis and healthy individuals showed that both lncRNAs and, to a lesser extent, short ncRNAs undergo significant changes during sepsis in healthy individuals (81). So, different types of ncRNAs can serve as potential biomarkers for sepsis and as new therapeutic targets (82).

## Potential Epigenetic Therapies for Sepsis

Several studies evaluated the potential therapeutic effect of epigenetic drugs in modulating chromatin regulatory enzymes during sepsis. Animal research has shown that epigenetic mechanisms can mitigate the acute inflammatory response to endotoxins (39, 48). Many of these epigenetic therapies are undergoing clinical trials to treat different cancers (83–85). Some of these therapies have been approved by the Food and Drug Administration and are used in clinical practice.

Suberoylanilide hydroxamic acid (SAHA) is a histone deacetylase (HDACi) inhibitor known for its anti-tumor effects. Leoni and colleagues showed that SAHA could also reduce the production of pro-inflammatory cytokines. Mice treated with this inhibitor reduced the production of TNF-α, IL-1β, IL-6, and interferon gamma (IFN-γ) after a challenge with LPS (60). When mice were submitted to the polymicrobial sepsis model initiated by ligation and cecal puncture (CLP) (61, 86), survival improved and damage reduced.

Another HDACi that appears to be effective in improving the clinical outcomes of sepsis is Trichostatin A (TSA) (86). TSA-pretreated mice submitted to CLP presented a protective effect during sepsis-induced lung injury, with reduced inflammatory infiltrate, decreased expression of the intercellular adhesion molecule-1 (ICAM-1) and E-selectin in lung tissue samples, and reduced plasma IL-6, with increased survival (57). Treatment with TSA, in combination with DNA methyltransferase inhibitor (DNMTi) 5-Aza 2-deoxycytidine (5-AZA-CdR), decreases apoptosis and inflammation in BMDMs of mice exposed to LPS (56). Also, TSA blocks the effect of endotoxin tolerance in reducing IL-6 production (87).

Valproic acid (VPA) and sodium butyrate (SB) also act as HDACi and have shown efficacy in experimental models of sepsis, with reduced expression of inflammatory genes and decreased organic damage (57, 88, 89); however, toxicity may prevent its use in clinical trials (90).

Studies show that different DNMTi can reverse some sepsis results in an endotoxemia model in rats. As an example, procainamide inhibited the increase in DNMT1 and decreased neutrophil infiltration in the lung of endotoxemic rats (59). Decitabine, a DNMTi, reduced NF-kB activation, decreased the levels of inflammatory cytokines, and inhibited sepsis progression in mice challenged with CLP (58).

The mechanisms by which DNMTi and HDACi act to reverse some of the consequences of sepsis are still not fully understood. These inhibitors are believed to prevent epigenetic changes and their modulating effects on gene expression (39). However, use of these inhibitors may become unfavorable because they reduce the expression of pro-inflammatory and anti-inflammatory cytokines and mediators, decreasing bacterial clearance (91).

Despite the effects of these enzymes' inhibitors in pre-clinical models of sepsis, one should be cautious to translate this approach as a potential clinical adjuvant therapy for sepsis. In most studies already mentioned here, the inhibitors were administered prophylactically, which does not mimic the setting of sepsis therapy. Few are those who demonstrate the benefits of late epigenetic drug use. One used the highly specific SIRT1 inhibitor, EX-527, in mice 24 h after onset of sepsis. All animals receiving this epigenetic agent survived sepsis with the reversion of endotoxin tolerance (92). Furthermore, other animal models should also be tested. For other potential uses of these drugs, the benefits must overcome the risks and toxicity.

## Conclusion

During sepsis, dysregulated gene expression occurs, generating hyperinflammatory responses and, in parallel, persistent hypo-inflammatory reactions. Strong evidence points to epigenetic changes as one of the main factors influencing gene expression changes associated with this clinical condition. In this review, we summarize studies that highlight epigenetic mechanisms as essential events during sepsis pathology, changing as sepsis progresses. Thus, epigenetic regulation occurs mainly in transcriptional promoter regions or gene enhancers, leading to pathological and bioenergetic adaptations through specific enzymes that catalyze chemical groups. The use of epigenetic enzyme inhibitors is promising as a therapeutic target during sepsis, but further research is needed to understand their role in clinical settings.

## Author Contributions

RF-H wrote the first draft of the article. RF-H and RS reviewed and wrote the final draft of article. MJ and MB contributed to paper revision and to the final version. All authors contributed to the article and approved the submitted version.

## Conflict of Interest

The authors declare that the research was conducted in the absence of any commercial or financial relationships that could be construed as a potential conflict of interest.
