# Supplementary material for: Epigenetic Regulation in Sepsis, Role in Pathophysiology and Therapeutic Perspective
Source: Front Med (Lausanne). 2021 Jul 12;8:685333. doi: 10.3389/fmed.2021.685333 (PMC8312749; doi:10.3389/fmed.2021.685333)
Supplement: Supplementary file 1 [file Table_1.DOCX]

**Abbreviations**

5mC: 5-methylcytosine;

5-AZA-CdR: 5-Aza 2’- deoxycytidine;

ALI: Acute lung injury;

BCG: Bacille Calmette-Guérin;

BET: Bromo- and extra-terminal;

BMDM: Bone marrow-derived macrophages;

CIITA: Class II transactivator;

CLP: Cecal ligation and puncture;

CLR: C-type lectin receptor;

DAMP: Damage-associated molecular pattern;

DC: Dendritic cell;

DMRs: Differentially methylated regions;

DNMT: DNA methyltransferase;

DNMTi: DNA methyltransferase inhibitor;

HAT: Histone acetyltransferase;

HDAC: Histone deacetylase;

HDACi: Histone deacetylase inhibitor;

HDM: Histone demethylase;

HLA: Human leukocyte antigen;

HMT: Histone methyltransferases;

ICAM-1: Intercellular adhesion molecule 1;

IFN-γ: Interferon gamma;

IL: Interleukin;

JMJD3: Jumonji domain-containing protein D3;

lncRNA: Long non-coding RNA;

LPS: Lipopolysaccharide;

MHC: Major histocompatibility complex;

mRNA: Messenger RNA;

miRNA: MicroRNA;

MODS: Multiple organ dysfunction syndrome;

NAD: Nicotinamide adenine dinucleotide;

ncRNA: Non-coding RNA;

NF-kB: Nuclear factor-κB;

NLR: NOD-like receptor;

Nt: Nucleotides;

PAMP: Pathogen-associated molecular pattern;

PcG: Polycomb group;

PTMs: Post-translational modifications;

PRR: Pattern recognition receptor;

RLR: RIG-I-like Receptor;

SAHA: Suberoylanilide Hydroxamic Acid;

SB: Sodium butyrate;

SIRT: Sirtuin;

TCA: Tricarboxylic acid;

TLR: Toll-like receptor;

TNF: Tumor necrosis factor;

TSA: Trichostatin A;

VPA: Valproic acid;

αKG: α-ketoglutarate.
